# Genome-wide genetic analyses highlight mitogen-activated protein kinase (MAPK) signaling in the pathogenesis of endometriosis

**DOI:** 10.1093/humrep/dex024

**Published:** 2017-02-09

**Authors:** Outi Uimari, Nilufer Rahmioglu, Dale R. Nyholt, Katy Vincent, Stacey A. Missmer, Christian Becker, Andrew P. Morris, Grant W. Montgomery, Krina T. Zondervan

**Affiliations:** 1 Endometriosis CaRe Centre, Nuffield Department of Obstetrics and Gynaecology, University of Oxford, Level 3, Women's Centre, John Radcliffe Hospital, OxfordOX3 9DU, UK; 2 Wellcome Trust Center for Human Genetics, University of Oxford, Roosevelt Drive, OxfordOX3 7BN, UK; 3 Institute of Health and Biomedical Innovation, Queensland University of Technology, 60 Musk Avenue, Kelvin Grove, QLD 4059, Australia; 4 Department of Obstetrics, Gynecology and Reproductive Biology, Brigham and Women's Hospital and Harvard Medical School, 75 Francis Street, Boston, MA 02115, USA; 5 Department of Biostatistics, University of Liverpool, LiverpoolL69 3GL, UK; 6 Institute for Molecular Bioscience, The University of Queensland, Brisbane, QLD 4072, Australia

**Keywords:** endometriosis, genetics, genome-wide association, pathway analysis, disease subtypes, MAPK signaling

## Abstract

**STUDY QUESTION:**

Do genome-wide association study (GWAS) data for endometriosis provide insight into novel biological pathways associated with its pathogenesis?

**SUMMARY ANSWER:**

GWAS analysis uncovered multiple pathways that are statistically enriched for genetic association signals, analysis of Stage A disease highlighted a novel variant in MAP3K4, while top pathways significantly associated with all endometriosis and Stage A disease included several mitogen-activated protein kinase (MAPK)-related pathways.

**WHAT IS KNOWN ALREADY:**

Endometriosis is a complex disease with an estimated heritability of 50%. To date, GWAS revealed 10 genomic regions associated with endometriosis, explaining <4% of heritability, while half of the heritability is estimated to be due to common risk variants. Pathway analyses combine the evidence of single variants into gene-based measures, leveraging the aggregate effect of variants in genes and uncovering biological pathways involved in disease pathogenesis.

**STUDY DESIGN, SIZE, DURATION:**

Pathway analysis was conducted utilizing the International Endogene Consortium GWAS data, comprising 3194 surgically confirmed endometriosis cases and 7060 controls of European ancestry with genotype data imputed up to 1000 Genomes Phase three reference panel. GWAS was performed for all endometriosis cases and for Stage A (revised American Fertility Society (rAFS) I/II, *n* = 1686) and B (rAFS III/IV, *n* = 1364) cases separately. The identified significant pathways were compared with pathways previously investigated in the literature through candidate association studies.

**PARTICIPANTS/MATERIALS, SETTING, METHODS:**

The most comprehensive biological pathway databases, MSigDB (including BioCarta, KEGG, PID, SA, SIG, ST and GO) and PANTHER were utilized to test for enrichment of genetic variants associated with endometriosis. Statistical enrichment analysis was performed using the MAGENTA (Meta-Analysis Gene-set Enrichment of variaNT Associations) software.

**MAIN RESULTS AND THE ROLE OF CHANCE:**

The first genome-wide association analysis for Stage A endometriosis revealed a novel locus, rs144240142 (*P* = 6.45 × 10^−8^, OR = 1.71, 95% CI = 1.23–2.37), an intronic single-nucleotide polymorphism (SNP) within *MAP3K4*. This SNP was not associated with Stage B disease (*P* = 0.086). *MAP3K4* was also shown to be differentially expressed in eutopic endometrium between Stage A endometriosis cases and controls (*P* = 3.8 × 10^−4^), but not with Stage B disease (*P* = 0.26). A total of 14 pathways enriched with genetic endometriosis associations were identified (false discovery rate (FDR)-*P* < 0.05). The pathways associated with any endometriosis were *Grb2-Sos provides linkage to MAPK signaling for integrins* pathway (*P* = 2.8 × 10^−5^, FDR-*P* = 3.0 × 10^−3^), *Wnt signaling* (*P* = 0.026, FDR-*P* = 0.026) and *p130Cas linkage to MAPK signaling for integrins* pathway (*P* = 6.0 × 10^−4^, FDR-*P* = 0.029); with Stage A endometriosis: extracellular signal-regulated kinase (*ERK)1 ERK2 MAPK* (*P* = 5.0 × 10^−4^, FDR-*P* = 5.0 × 10^−4^) and with Stage B endometriosis: two overlapping pathways that related to extracellular matrix biology—*Core matrisome* (*P* = 1.4 × 10^−3^, FDR-*P* = 0.013) and *ECM glycoproteins* (*P* = 1.8 × 10^−3^, FDR-*P* = 7.1 × 10^−3^). Genes arising from endometriosis candidate gene studies performed to date were enriched for *Interleukin signaling pathway* (*P* = 2.3 × 10^−12^), *Apoptosis signaling pathway* (*P* = 9.7 × 10^−9^) and *Gonadotropin releasing hormone receptor pathway* (*P* = 1.2 × 10^−6^); however, these pathways did not feature in the results based on GWAS data.

**LARGE SCALE DATA:**

Not applicable.

**LIMITATIONS, REASONS FOR CAUTION:**

The analysis is restricted to (i) variants in/near genes that can be assigned to pathways, excluding intergenic variants; (ii) the gene-based pathway definition as registered in the databases; (iii) women of European ancestry.

**WIDER IMPLICATIONS OF THE FINDINGS:**

The top ranked pathways associated with overall and Stage A endometriosis in particular involve integrin-mediated *MAPK* activation and intracellular *ERK/MAPK* acting downstream in the *MAPK* cascade, both acting in the control of cell division, gene expression, cell movement and survival. Other top enriched pathways in Stage B disease include *ECM glycoprotein* pathways important for extracellular structure and biochemical support. The results highlight the need for increased efforts to understand the functional role of these pathways in endometriosis pathogenesis, including the investigation of the biological effects of the genetic variants on downstream molecular processes in tissue relevant to endometriosis. Additionally, our results offer further support for the hypothesis of at least partially distinct causal pathophysiology for minimal/mild (rAFS I/II) vs. moderate/severe (rAFS III/IV) endometriosis.

**STUDY FUNDING/COMPETING INTEREST(S):**

The genome-wide association data and Wellcome Trust Case Control Consortium (WTCCC) were generated through funding from the Wellcome Trust (WT084766/Z/08/Z, 076113 and 085475) and the National Health and Medical Research Council (NHMRC) of Australia (241944, 339462, 389927, 389875, 389891, 389892, 389938, 443036, 442915, 442981, 496610, 496739, 552485 and 552498). N.R. was funded by a grant from the Medical Research Council UK (MR/K011480/1). A.P.M. is a Wellcome Trust Senior Fellow in Basic Biomedical Science (grant WT098017). All authors declare there are no conflicts of interest.

## Introduction

Endometriosis is a chronic inflammatory disease in which endometrial-like tissue is located outside the uterus. It is associated with debilitating pelvic pain and reduced fertility, and affects 1.5 M women in the UK (176 M worldwide) (Simoens *et al*., 2012). Treatments are limited to surgical removal of disease tissue, and hormonal drugs with many side effects. Identifying novel diagnostic tools and treatments requires a better understanding of the pathogenesis of endometriosis. Although advances have certainly been made in the past decade in understanding of biological processes likely to promote the maintenance and growth of lesions, the actual causes remain largely unclear. Previous investigations have considered the potential roles of steroids, endometrial aberrations, altered peritoneal environment, reduced immune surveillance and increased angiogenic capacity in the pathogenesis (Giudice and Kao, 2004) but, crucially, it is not known whether these processes are a necessary cause, or rather an effect of the disease process.

One method to improve understanding of complex disease causality is through the identification of genetic factors underlying disease risk, through genome-wide association studies (GWAS). The involvement of genetic factors in the development of endometriosis is supported by numerous studies (Simpson, *et al*., 1980; Bischoff and Simpson, 2004; Rahmioglu *et al*., 2015a, 2015b), and its heritability is estimated at ~50% (Treloar *et al*., 1999; Saha *et al*., 2015). Before the advent of GWAS, candidate gene association studies were the most common type of study in the investigation of genetic factors underlying complex diseases, and many such studies have been published for endometriosis (Rahmioglu, *et al*., 2012; Montgomery *et al*., 2014). Results from candidate association studies have been poorly replicated for a number of reasons that are intrinsic to their design (Rahmioglu *et al*., 2015b). GWAS bypasses the weaknesses of candidate association studies as they are not based on an *a priori* biological hypothesis.

The GWAS for endometriosis conducted to date have identified 10 genome-wide significantly associated variants (Rahmioglu *et al*., 2014; Zondervan *et al*., 2016), in data sets including women of European and Japanese ancestry (Uno *et al*., 2010; Painter *et al*., 2011; Nyholt *et al*., 2012; Albertsen *et al*., 2013; Sapkota *et al*., 2015). Most of these show stronger association with moderate/severe disease, but together they explain <4% of heritability (Rahmioglu *et al*., 2014; Zondervan *et al*., 2016). Of the total variance in disease risk, 24% has been estimated to be due to common genetic variants (Lee *et al*., 2013). Larger GWAS meta-analyses are required to bring the field of endometriosis up to speed with progress made in other areas (e.g. breast cancer: 67 common genetic predisposing loci are now known, from meta-analysis of >55 000 cases; Michailidou *et al*., 2013); however, other approaches are also needed to identify remaining genetic variants and pathways.

One disadvantage of GWAS is the requirement for a very stringent genome-wide significance threshold (*P* < 5 × 10^−8^) to identify variants likely to be genuinely associated with a complex disease. Such a stringent threshold is necessary because of the large number of statistical tests being conducted when testing each individual variant for association with the disease; however, it results in true associations not reaching this threshold being missed. One approach to uncover genetic variants associated with the disease under this multiple-testing burden is to aggregate genetic variants passing a pre-defined significance threshold for association with a disease into gene-based measures and to investigate whether the number of associated genes in a given pathway is greater than expected by chance (Perry *et al*., 2009).

We aimed to explore whether the largest GWAS data set to date comprising 3194 surgically confirmed endometriosis cases (Painter *et al*., 2011) can provide insight into novel biological pathways causally involved in endometriosis pathogenesis. We tested pathways, without any preselection, for evidence of enrichment of genetic variants associated with endometriosis. We also performed the first GWAS analysis for minimal/mild (Stage A) endometriosis. Lastly, we investigated to what extent pathways that have been investigated most frequently through candidate gene association studies feature in those highlighted in GWAS-based pathway analyses.

## Materials and Methods

### GWAS participants and phenotyping

The International Endogene Consortium (IEC) GWAS data included 3194 surgically confirmed endometriosis cases, and 7060 controls of European ancestry from Australia and the UK (Supplementary Table SI) (Painter *et al*., 2011). Cases were classified according to the revised American Fertility Society (rAFS) classification for endometriosis severity through assessment of surgical records and grouped into two sub-phenotypes: Stage A (rAFS Stage I or II, or some ovarian disease with a few adhesions: peritoneal or superficial ovarian disease with filmy adhesions, *n* = 1686) and Stage B (rAFS Stage III–IV: deep ovarian and/or rectovaginal disease with dense adhesions, *n* = 1364) (ASRM, 1985). Australian controls included 1870 parents and siblings of adolescent twins recruited as part of the Brisbane Adolescent Twin Study. The UK controls included 3000 individuals from the 1958 British Birth Cohort and 3000 from National Blood Service Donors, provided by the Wellcome Trust Case Control Consortium 2 (Wellcome Trust Case Control *et al*., 2007, 2010).

### Genotyping and imputation

Cases were genotyped using the Illumina Human670Quad BeadArray, the Australian controls on Illumina Human610Quad and the UK controls on Illumina Human1M-Duo. The genotype data, including the autosomes and chromosome X, were imputed to the latest 1000 Genomes Phase three reference panel (October 2014). Pre-phasing and imputation were performed using SHAPEIT2 (Delaneau *et al*., 2014) and IMPUTE2 (Howie *et al*., 2009) softwares, respectively.

### Genome-wide association and functional analyses

Three sets of genome-wide association analysis were performed: (i) All cases (*n* = 3194), (ii) Stage A cases (*n* = 1686) and (iii) Stage B cases (*n* = 1364) vs. controls (*n* = 7060) to see whether the two sub-phenotypes share similar underlying or have distinct underlying genetic factors in their causation. Logistic regression analysis including a covariate representing the Australian and the UK strata was performed in SNPTESTv2 (Marchini *et al*., 2007). After genome-wide association analysis, the results from biallelic single-nucleotide polymorphisms/(INsertion/DELetion) (SNPs/INDELs) with imputation quality >0.8 and minor allele frequency (MAF) >0.01 were retained (*n* = 8 943 157). QQ plots for all cases, Stage A and Stage B disease associations are provided in Supplementary Figure S1.

Genetic variants associated with overall, Stage A and Stage B disease (*P* < 1 × 10^−6^) were checked for functional evidence in the genomic region in the Encyclopedia of DNA Elements (ENCODE) consortium data (Consortium, 2012). Histone modification marks were identified from seven cell lines (GM12878, H1-hESC, HSMM, HUVEC, K562, NHEK, NHLF) and DNAse I hypersensitivity peaks identified from 95 cell types from ENCODE database using UCSC (University of California, Santa Cruz) genome browser annotation tools (endometrial tissue limited to four samples only).

The genes closest to the genetic variants associated with overall, Stage A and Stage B disease (*P* < 1 × 10^−6^) were tested for differential expression using a previously published, publicly available data set (GEO Accession: GSE51981) of eutopic endometrium from 77 endometriosis cases (Stage A *n* = 27, Stage B *n* = 48, unclassified *n* = 2) and 71 endometriosis-free controls assayed using the Affymetrix Human Genome U133 Plus 2.0 Array (Tamaresis *et al*., 2014). Comparisons between groups to identify differentially expressed genes were conducted on original submitter-supplied processed data using GEOquery and limma R packages in GEO2R (Smyth, 2005; Davis and Meltzer, 2007). We performed differential expression analysis including all cases vs. controls from all menstrual phases, checked whether the most significant probes per gene region were differentially expressed between menstrual phases, and if so, conducted within-phase differential expression analysis between cases and controls.

### Pathway analysis using GWAS results in MAGENTA

Pathway genetic enrichment analysis was performed for (i) all endometriosis, (ii) Stage A endometriosis, (iii) Stage B endometriosis GWAS results, using MAGENTA (Meta-Analysis Gene-set Enrichment of variaNT Associations) software (Segre *et al*., 2010) (Supplementary Figure S2). MAGENTA first maps SNPs to genes taking 110 Kb upstream and 40 Kb downstream of each gene as extended boundaries to include regulatory regions. Each gene is then assigned a genetic score (GS), which is the *P*-value of the most significant SNP within the gene's extended boundaries, corrected for six potential confounding factors of physical and genetic properties of genes through a step-wise multiple linear regression: (i) the physical size of the gene, (ii) number of SNPs per kilobase for each gene, (iii) estimated number of independent SNPs per gene, (iv) number of recombination hotspots spanning each gene, (v) genetic distance of the gene and (vi) linkage disequilibrium (LD) unit distance per gene.

We built a pathway database library from the two most comprehensive and up-to-date resources, Molecular Signatures database v5.1 (MSigDB) and PANTHER pathway database v10.0 (15 December 2015), and used this library in the pathway genetic enrichment analyses (Supplementary Figure S2). The Molecular Signatures Database (MSigDB) version 5.1 (Mootha *et al*., 2003; Subramanian *et al*., 2005) (http://software.broadinstitute.org/gsea/msigdb/index.jsp) is the most extensive resource of gene sets/pathways, currently comprising 13 311 non-independent gene sets. From this database, we downloaded (i) the curated canonical pathways (C2-CP), which include 1330 gene sets amalgamated from BioCarta, Kyoto Encyclopedia of Genes and Genomes (KEGG), Matrisome, Pathway Interaction Database (PID), Reactome, SigmaAldrich (SA), Signaling Gateway (SIG), Signaling Transduction KE (ST) and SuperArray and (ii) Gene Ontology (GO) gene sets, which include 1454 gene sets comprised of biological processes, cellular components, molecular functions. We also downloaded 149 curated canonical pathways from the PANTHER database version 10.0 (Mi *et al*., 2016), which were not part of the MSigDB. The total number of pathways included was 2933, with the effective number tested *n* = 2860 (excluding pathways with <10 genes) (Supplementary Figure S2).

MAGENTA employs a Gene Set Enrichment Analysis (GSEA)-like statistic (Mootha *et al*., 2003; Subramanian *et al*., 2005) to the gene association *P*-values adjusted for confounding factors. The null hypothesis in the analysis is that the tested genes are randomly distributed in terms of significance (*P*-values) of association with the disease of interest, within each pathway. The alternative hypothesis assumes enrichment above a given rank cutoff compared with multiple random gene sets. The rank cutoff is a pre-determined gene *P*-value cutoff, and is defined as a given percentile of all gene *P*-values in the genome. We used a 95% cutoff threshold for significance. Multiple-testing correction was applied through calculation of the false discovery rate (FDR) for resources with >25 genesets/pathways. Results from smaller resources with <25 pathways, which were more likely to be independent of each other, were also adjusted using a Bonferroni correction (*P* = 0.05/number of pathways tested). The software was obtained from http://www.broadinstitute.org/mpg/magenta/ and ran locally in MATLAB R2013a.

### Identification of pathways suggested by previously published candidate gene studies

The 122 candidate genes (Rahmioglu *et al*., 2012) that have been investigated for association with endometriosis were queried using the PANTHER 10.0 database on 15 December 2015, which contains pathway information on 20 000 genes within 149 curated human pathways. The genes were tested for over-representation in these pathways (Mi *et al*., 2016) by calculating the difference between the observed fraction of genes in that pathway and the number expected by chance, with significance of over-representation tested using a Fisher's exact test. The PANTHER pathways that showed statistically significant (*P* < 0.05 after Bonferroni multiple-testing correction) over-representation of the candidate genes were defined as the pathways that have been investigated indirectly through candidate gene association studies (Supplementary Figure S2).

## Results

After imputation to the latest 1000 Genomes Phase three reference panel, no novel genome-wide significant associations with overall endometriosis (*n* = 3194) or Stage B (*n* = 1364) (Table I, Supplementary Figure S3) were observed; the well-established rs12700667 remained the strongest association (OR = 1.32, 95% CI 1.20–1.46; *P* = 2.45 × 10^−9^ with Stage B disease). The second genome-wide significant locus remained at *FN1*, though imputation altered the strongest associated SNP from the previously reported rs1250258 (Painter *et al*., 2011) to highly correlated (*r*^2^ = 0.96) rs1250248 (OR = 0.81, 95% CI 0.74–0.88; *P* = 3.48 × 10^−8^). We found 11 loci (Table I) showing nominal association (*P* < 1.0 × 10^−6^), 3 of which were established endometriosis loci (*WNT4, CDKN2BAS1, ID4*) and 8 had not previously been reported. Of these eight loci, three were associated with all endometriosis (*LAMC3*, *CAPN14* and *DEFA1*), and six with Stage B disease (*NAALADL2, NR2C1, C14orf132, FOXP2, CDH20* and *LAMC3*).

**Table I:** Independent signals from overall, Stage A and Stage B GWAS results with *P* < 1 × 10^−6^.

Rsid (Chr:Position)	A1/A2 (MAF)	Overall *P*	Overall OR (95% CI)	Stage A *P*	Stage A OR (95% CI)	Stage B *P*	Stage B OR (95% CI)	Variant type	Nearest gene (distance)	Regulatory function from ENCODE (±25 Kb)*
*Overall endometriosis GWAS*										
rs6908034 (6:19773930)	G/A (0.16)	5.36 × 10^−7^	1.22 (1.12–1.32)	0.012	1.13 (1.02–1.25)	7.31 × 10^−7^	1.30 (1.17–1.45)	Intronic SNP	ID4 (64 056bp)	Located in an anti-sense RNA, RP1-167F1.2
rs12700667 (7:25901639)	G/A (0.25)	5.57 × 10^−7^	1.17 (1.09–1.25)	0.038	1.07 (0.98–1.16)	2.45 × 10^−9^	1.32 (1.20–1.46)	Intergenic SNP	NFE2L3 (290 221bp)	(1) Near a microRNA, mir148a (87 900bp), (2) In histone modification marks H3K27AC, H3K4Me1, H3K4Me3, (3) In/near TFB sites for MXI1, POLR2A, TBP, NFYA, ARID3A, GATA3, ELF1, TEAD4, JUND, SMARCA4, SIX5, MAX, NRF1, RFX5, CHD2, CREB1, CEBPC, ATF1, KDM5B, JUN, NFYB, RUNX3, SP4, MAZ, SIN3A, ZBTB7A, MYC, STAT3, HMGN3, CCNT2, CBX3, TCF3, BHLHE40, EP300, E2F6, FOXP2, GABPA, ZNF143, SPI1, USF1, EGR1, E2F4, E2F1, MAFK, TCF7L2, POU2F2, TAF1, PHF8, IRF1, FOXA1 (±1 Kb)
rs55938609 (1:22470451)	G/C (0.16)	6.11 × 10^−7^	1.24 (1.15–1.35)	2.36 × 10^−3^	1.17 (1.06–1.29)	8.33 × 10^−7^	1.33 (1.20–1.48)	SNP Upstream of gene	WNT4 (932bp)	(1) In histone modification mark H3K4Me1, (2) In/near TFB sites for EZH2, FOXA2, FOXA1, EZH2, RAD21, CTCF, PAX5, POLR2A, E2F1, EGR1, CCNT2, SIN3A, RBBP5 (±1 Kb)
rs138913144 (9:133897939)	A/ATATT (0.07)	6.38 × 10^−7^	0.77 (0.69–0.87)	4.47 × 10^−3^	0.86 (0.74–1.00)	9.94 × 10^−7^	0.68 (0.57–0.81)	Intronic Insertion	LAMC3 (0bp)	(1) Near a small nucleolar RNA, SNORA31 (1246bp), (2) Near histone modification mark H3K4Me1
rs116175374 (2:31425185)	G/A (0.04)	6.45 × 10^−7^	0.69 (0.59–0.81)	4.03 × 10^−5^	0.68 (0.55–0.85)	1.79 × 10^−3^	0.73 (0.58–0.92)	Intronic SNP	CAPN14 (0bp)	(1) Near histone modification mark H3K4Me1
rs60966186 (8:6831204)	A/G (0.19)	9.85 × 10^−7^	0.85 (0.79–0.92)	6.71 × 10^−5^	0.87 (0.79–0.96)	3.62 × 10^−5^	0.84 (0.75–0.93)	Intergenic SNP	DEFA1 (4088bp)	(1) Near histone modification mark H3K4Me1, (2) Near TFB site for KAP1 (±1 Kb)
*Stage A endometriosis GWAS*										
rs144240142 (6:161503024)	T/C (0.01)	5.79 × 10^−5^	1.46 (1.10–1.93)	6.45 × 10^−8^	1.71 (1.23–2.37)	0.086	1.19 (0.79–1.78)	Intronic SNP	MAP3K4 (0bp)	(1) Transcribed on seven cell lines assayed by RNA-seq data, (2) Near TFB sites for EGR1, CEBPB, FOSL1, FOS (±1 Kb)
rs200922190 (1:193203491)	A/AAATTAT (0.22)	2.35 × 10^−4^	0.90 (0.84–0.97)	1.79 × 10^−7^	0.83 (0.75–0.91)	0.097	0.97 (0.88–1.07)	Intronic Insertion	CDC73 (0bp)	(1) Transcribed on seven cell lines assayed by RNA-seq data, (2) Near TFB sites for TCF7L2, SETDB1, FOXA1, KAP1, TFAP2A, TFAP2C, FOS, ELF1, FAM48A (±1 Kb).(3) Near histone modification mark H3K4Me1, H3K27Ac
8:2806920	G/GAAAGAAAAGAAAAGAAAAG (0.17)	0.73	1.36 (0.43–4.30)	3.82 × 10^−7^	0.79 (0.71–0.88)	0.14	0.94 (0.84–1.05)	Intronic deletion	CSMD1 (0bp)	(1) Near histone modification mark H3K4Me1, (2) Near TFB sites for MAX, FOXP2, REST.
rs855965 (10:119443759)	G/A (0.34)	1.23 × 10^−3^	0.89 (0.84–0.95)	4.10 × 10^−7^	0.81 (0.74–0.88)	0.96	1.00 (0.92–1.09)	Intergenic SNP	EMX2 (1 34 702bp)	(1) Near histone modification mark H3K4Me1, (2) Near TFB sites for STAT3, CTCF, RAD21, SMC3, ESR1
rs113850637 (3:103850400)	C/T (0.16)	4.56 × 10^−4^	1.15 (1.06–1.25)	8.08 × 10^−7^	1.28 (1.16–1.41)	0.60	1.01 (0.90–1.13)	Intergenic SNP	ALCAM (1 235 853bp)	(1) Near a microRNA, mir548a3 (53076), (2) Near histone modification mark H3K4Me1, (3) Near TFB sites for EP300, GATA2, JUN, FOS, MAX, USF1, YY1, CTCF, TCF12, POU5F1
*Stage B endometriosis GWAS*										
rs12700667 (7:25901639)	G/A (0.25)	5.57 × 10^−7^	1.17 (1.09–1.25)	0.038	1.07 (0.98–1.16)	2.45 × 10^−9^	1.32 (1.20–1.46)	Intergenic SNP	NFE2L3 (290 221bp)	(1) Near a microRNA, mir148a (87900 bp),(2) In histone modification marks H3K27AC, H3K4Me1, H3K4Me3, (3) In/near TFB sites for MXI1, POLR2A, TBP, NFYA, ARID3A, GATA3, ELF1, TEAD4, JUND, SMARCA4, SIX5, MAX, NRF1, RFX5, CHD2, CREB1, CEBPC, ATF1, KDM5B, JUN, NFYB, RUNX3, SP4, MAZ, SIN3A, ZBTB7A, MYC, STAT3, HMGN3, CCNT2, CBX3, TCF3, BHLHE40, EP300, E2F6, FOXP2, GABPA, ZNF143, SPI1, USF1, EGR1, E2F4, E2F1, MAFK, TCF7L2, POU2F2, TAF1, PHF8, IRF1, FOXA1 (±1 Kb)
rs517875 (3:174350886)	C/A (0.42)	5.56 × 10^−4^	1.09 (1.03–1.16)	0.35	1.01 (0.93–1.09)	1.06 × 10^−7^	1.23 (1.13–1.33)	Intronic SNP	NAALADL2 (0bp)	(1) Near TFB sites for MAFK, ESR1
rs7041895 (9:22162794)	A/C (0.43)	4.81 × 10^−4^	1.12 (1.05–1.18)	0.60	1.02 (0.95–1.10)	1.06 × 10^−7^	1.26 (1.16–1.37)	Intergenic SNP	CDKN2BAS1 (41 701bp)	Near histone modification mark H3K4Me1
rs1250258 (2:216300185)	C/T (0.27)	2.48 × 10^−5^	0.89 (0.83–0.95)	0.22	0.98 (0.90–1.07)	3.48 × 10^−8^	0.81 (0.74–0.88)	Intronic SNP	FN1 (0bp)	(1) In histone modification mark H3K4Me3, H3K27Ac. (2) In/near TFB sites for POLR2A, TEAD4, TAF1, MBD4, MXI1, RBBP5, SIN3A, FOXA2, MAX, EZH2, RCOR1, MYC
12:95403979 (12:95403979)	C/CT (0.11)	3.35 × 10^−6^	1.24 (1.13–1.36)	0.13	1.11 (0.98–1.24)	2.43 × 10^−7^	1.38 (1.23–1.56)	Intergenic SNP	NR2C1 (12 026bp)	(1) Near TFB sites for TRIM28, CBX3, USF1, CTCF
rs71415016 (14:96443958)	T/C (0.08)	1.16 × 10^−4^	1.19 (1.06–1.32)	0.16	1.03 (0.89–1.19)	3.00 × 10^−7^	1.38 (1.20–1.59)	Intergenic SNP	C14orf132 (61 880bp)	Near histone modification mark H3K4Me1
rs62469231 (7:114031174)	G/A (0.02)	1.10 × 10^−5^	1.44 (1.18–1.77)	0.018	1.23 (0.94–1.60)	4.95 × 10^−7^	1.77 (1.37–2.27)	Intronic SNP	FOXP2 (0bp)	Near histone modification mark H3K4Me1
rs3920498 (1:22492887)	G/C (0.20)	1.10 × 10^−5^	1.19 (1.11–1.28)	0.034	1.11 (1.02–1.22)	6.49 × 10^−7^	1.30 (1.18–1.43)	Intergenic SNP	WNT4 (22 502bp)	(1) In histone modification mark H3K4Me1, (2) In/near TFB sites for RELA, pouf2f2, ebf1 (±1 Kb).
rs6908034 (6:19773930)	G/A (0.16)			0.012	1.13 (1.02–1.25)	7.31 × 10^−7^	1.30 (1.17–1.45)	Intronic SNP	ID4 (64 056bp)	Located in an anti-sense RNA, RP1-167F1.2
rs12455952 (18:58840518)	T/G (0.19)	1.75 × 10^−5^	1.16 (1.07–1.25)	0.021	1.09 (0.99–1.20)	9.59 × 10^−7^	1.27 (1.15–1.40)	Intergenic SNP	CDH20 (317 287bp)	Near TFB sites for MAFK, E2F4, FOS
rs138913144 (9:133897939)	A/ATATT (0.07)			4.47 × 10^−3^	0.86 (0.74–1.00)	9.94 × 10^−7^	0.68 (0.57–0.81)	Intronic insertion	LAMC3 (0bp)	(1) Near a small nucleolar RNA, SNORA31 (1246bp), (2) Near histone modification mark H3K4Me1

TFB sites, transcription factor binding sites; rsid, SNP ID; Chr, chromosome; MAF, minor allele frequency; *P,* Association test *P*-value, OR, odds ratio; SNP, single-nucleotide polymorphism; GWAS, genome-wide association study. *Histone modification marks as identified from seven cell lines (GM12878, H1-hESC, HSMM, HUVEC, K562, NHEK, NHLF) from ENCODE database and DNAse I hypersensitivity peaks identified from 95 cell types from ENCODE database using UCSC genome browser annotation tools.

**Figure 1:**
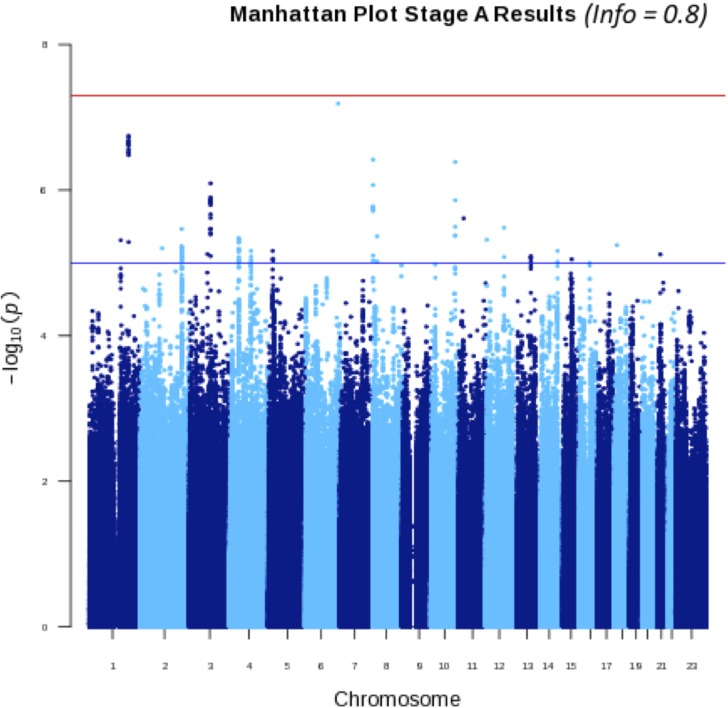
Manhattan plot of association of single-nucleotide polymorphisms (SNPs) with Stage A endometriosis in the GWAS. Red horizontal line marks the genome-wide significance (*P* < 5 × 10^−8^), and blue line marks nominal significance (*P* < 5 × 10^−6^).

Our first ever GWAS analysis for Stage A endometriosis (*n* = 1686 cases) revealed a novel locus, rs144240142 (*P* = 6.45 × 10^−8^, OR 1.71, 95%CI 1.23–2.37), an intronic SNP with MAF of 0.01, imputation info score >0.8, within the *MAP3K4* gene (Table I, Fig. 1). In addition, four more loci were associated with Stage A disease with nominal significance (*P* < 1.0 × 10^−6^): two INDELS within *CDC73* and *CSMD1* genes, and two intergenic SNPs near *EMX2* and *ALCAM* genes (Table I).

For each of the loci, we explored potential functionality using ENCODE (the Encyclopedia Of DNA Elements) Project data (Consortium, 2012) (see Materials and Methods). These showed most endometriosis-associated SNPs to be located near elements of the genome that are designated to be regulatory in a variety of cell types (Table I). The cell lines in which the regulatory elements were found included GM12878 (lymphoblastoid cell line), H1-hESC (human embryonic stem cells), HSMM (human skeletal muscle myoblasts), HUVEC (human umbilical vein endothelial cells), K562 (immortalized cell line produced from a female patient with chronic myelogenous leukemia), NHEK (normal human epidermal keratinocytes) and NHLF (normal human lung fibroblasts) (Consortium, 2012). The strongest associated endometriosis locus, rs12700667 (*P* = 2.45 × 10^−9^), is located in known epigenetic regulators (histone modification marks H3K27ac, H3K4Me1 and H3K4Me3), in/near transcription factor binding (TFB) sites for a number of genes, and near a microRNA (miR148a, 87.9 Kb away); the nearest gene is *NFE2L3* (Nuclear Factor, Erythroid 2-Like 3), located 290.2 Kb away (Table I). *MAP3K4* is transcribed in all seven cell lines (ENCODE RNA sequencing data) while rs144240142 is located near (±1 kb) TFB sites for *EGR1, CEBPB, FOSL1* and *FOS.*

We performed differential expression analysis for each of the genetic loci, using the largest publicly available eutopic endometrium microarray expression data set (Tamaresis *et al*., 2014) (see Materials and Methods). Differential expression between 77 cases vs. 71 controls was observed for *ID4, NFE2L3, MAP3K4, CDC73, CSMD1, FN1, NR2C1*and *C14orf132* (FDR-corrected *P* < 0.05). Of these, *ID4, NFE2L3*and* MAP3K4* were also differentially expressed between proliferative, early and mid secretory phases. Differential expression analysis within each phase for these loci showed that *ID4* and *MAP3K4* were significantly differentially expressed between cases and controls in the proliferative phase, while *NFE2L3* was not differentially expressed between cases and controls in any phase. All the loci associated with Stage A disease in our GWAS (*MAP3K4, CDC73, EMX2, ALCAM*) were more strongly differentially expressed when the analysis of the expression data set was restricted to Stage I/II cases vs. controls (27 cases vs. 71 controls). For Stage B associated loci, *FN1, NR2C1, C14orf132, NFE2L3,* we similarly observed stronger evidence of differential expression when analysis was restricted to Stage III/IV cases vs. controls (48 cases vs. 71 controls) (Supplementary Table SII).

**Figure 2:**
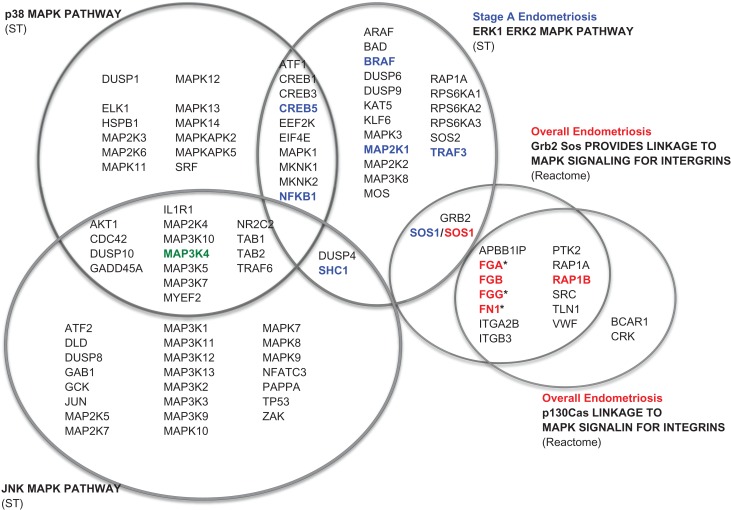
Mitogen-activated protein kinase (MAPK)-related pathways enriched for genome-wide association study (GWAS) associations with endometriosis.

Table II shows the results of the pathways that were significantly over-represented (‘enriched’) in the GWAS results, after correction for multiple testing (number of pathways; FDR-*P* < 0.05 or Bonferroni-*P* < 0.05 (see Materials and Methods)). *WNT* signaling—a key pathway implicated in our previous GWAS and pathway analyses—was significantly associated with all endometriosis at FDR < 0.05 but not after stringent Bonferroni correction taking account of the number of pathways in the SA database (see Materials and Methods). The top pathways significantly associated with all and Stage A endometriosis were mitogen-activated protein kinases (*MAPK*)-related, and originated from the large REACTOME database (Table II). *Grb2-Sos provides linkage to MAPK signaling for integrins* (all endometriosis, enrichment *P* = 2.8 × 10^−5^, FDR-*P* = 3.0 × 10^−3^) is a pathway that includes 15 genes, involving integrin clustering by fibronectin stimulation, linked to extracellular signal-regulated kinase (ERK)2 MAPK signaling. The genes enriched for association with endometriosis (*P* < 0.05) in this pathway were (i) *FN1* (fibronectin 1, *P* = 9.75 × 10^−4^); (ii) *FGA* (fibrinogen alpha chain, *P* = 1.95 × 10^−3^); (iii) *FGB* (fibrinogen beta chain; *P* = 3.95 × 10^−3^); (iv) *FGG* (fibrinogen gamma chain; *P* = 1.95 × 10^−3^); (v) *RAP1B* (Ras-related protein Rap-1b; *P* = 9.67 × 10^−3^) and (vi) *SOS1* (Son of sevenless homolog 1; *P* = 7.50 × 10^−4^). The second *MAPK*-related pathway significantly enriched for association with all endometriosis (*P* = 6.0 × 10^−4^, FDR-*P* = 0.029; Table II) was *P130Cas linkage to MAPK signaling for integrins*, a Reactome pathway also including 15 genes. This pathway signifies the role of p130Cas in survival signals and cell motility from the extracellular matrix (ECM) through integrin, activating the *ERK1 ERK2 MAPK* pathway. The two *MAPK*-related pathways define similar functions as they overlap by 12 genes (Fig. 2). Indeed, the top endometriosis-associated genes—shared by both pathways—were *FN1*, *FGA*, *FGB*, *FGG* and *RAP1B*.

Genes for which association contributing most to the enrichment (*P* < 0.05) are highlighted in bold. *Genes taking part in ECM glycoproteins and CORE matrisome pathways (NABA).

**Table II:** Genome-wide pathway analysis of all, Stage A and Stage B endometriosis results identified in MAGENTA.

Database (#pathways)	Pathway	Geneset size	*P*-value	FDR *P*-value	Bonferroni *P*-value*	Expected *N* genes	Observed *N* genes
*Overall endometriosis*							
REACTOME (671)	Grb2-Sos provides linkage to MAPK signaling for integrins	15	2.8 × 10^−5^	3 × 10^−3^	NA	1	6 (*FN1, FGG, FGA, FGB, RAP1B, SOS1*)
SA (9)	Wnt signaling	89	0.026	0.026	0.24	4	9 (*WNT4, CTNNBIP1, RHOU, PPP2R1A, CTBP2, HPRT1, CXXC4, SFRP1, KREMEN1*)
REACTOME (671)	p130CAS linkage to MAPK signaling for integrins	15	6 × 10^−4^	0.029	NA	1	5 (*FN1, FGG, FGA, FGB, RAP1B*)
*Stage A endometriosis*							
ST (23)	ERK1 ERK2 MAPK pathway	32	5 × 10^−4^	5 × 10^−4^	0.011	2	7 (*TRAF3, MAP2K1, SOS1, CREB5, NFKB1, SHC1, BRAF*)
SA (9)	TRKA receptor	17	1 × 10^−3^	2.5 × 10^−3^	9.0 × 10^−3^	1	5 (*ELK1, MAP2K1, SOS1, NTRK1, SHC1*)
SIG (8)	PIP3 signaling in cardiac myocytes	67	1.6 × 10^−3^	4.5 × 10^−3^	0.013	3	10 (*RPS6KB1, PTK2, YWHAG, PAK7, SOS1, CREB5, SHC1, CDKN1B, IGFBP1, GSK3B*)
ST (23)	G alpha S pathway	16	5.5 × 10^−3^	8.8 × 10^−3^	0.13	1	4 (*RASGRF2, CREB5, BRAF, SNX13*)
ST (23)	Phosphoinositide three kinase pathway	37	9.1 × 10^−3^	9.1 × 10^−3^	0.21	2	6 (*RPS6KB1, YWHAG, SOS1, SHC1, IGFBP1, GSK3B*)
ST (23)	Differentiation pathway in PC12 cells	45	5.5 × 10^−3^	0.019	0.13	2	7 (*CREBBP, ELK1, RASGRF2, CREB5, NTRK1, SHC1, BRAF*)
SA (9)	B cell receptor complexes	24	0.026	0.026	0.24	1	4 (*ELK1, MAP2K1, SOS1, SHC1*)
SIG (8)	Insulin receptor pathway in cardiac myocytes	51	0.033	0.033	0.26	2	6 (*RPS6KB1, YWHAG, SOS1, SHC1, IGFBP1, GSK3B*)
SA (9)	PTEN pathway	17	0.051	0.042	0.46	1	3 (*SOS1, IPCEF1, SHC1*)
*Stage B endometriosis*							
NABA (10)	ECM glycoproteins	196	1.8 × 10^−3^	7.1 × 10^−3^	0.018	9	19 (*FN1, LAMC3, THBS1, TNFAIP6, RSPO3, FBLN2, SPARC, VWF, GLDN, LAMB3, MXRA5, FGG, FGA, EMILIN2, TSPEAR, ZPLD1, EDIL3, WISP3, IGFBP6*)
NABA (10)	CORE Matrisome	275	1.4 × 10^−3^	0.013	0.014	13	24 (*FN1, LAMC3, THBS1, HSPG2, TNFAIP6, RSPO3, FBLN2, SPARC, SPOCK3, VWF, COL12A1, GLDN, LAMB3, MXRA5, DCN, LUM, FGG, FGA, EMILIN2, TSPEAR, ZPLD1, HAPLN4, KERA, EDIL3*)

*For databases with <25 pathways, we computed the Bonferroni *P*-value adjusted for the number of pathways within the given database resource, in addition to the FDR multiple-testing correction computed by the MAGENTA software (see Materials and Methods). Note that the Bonferroni adjustment is likely to be conservative given that the pathways within a resource are never fully independent of each other. Databases; SigmaAldrich (SA), Signaling Gateway (SIG), Signaling Transduction KE (ST), Matrisome Project gene sets (http://web.mit.edu/hyneslab/matrisome/) (Naba *et al*., 2012) (NABA).

For Stage A endometriosis, the top pathway enriched in the GWAS results was the *ERK1 ERK2 MAPK pathway* from the Signal Transduction (ST) database (*P* = 5.0 × 10^−4^, FDR-*P* = 5.0 × 10^−4^, Bonferroni-*P* = 0.011). The top associated genes in this pathway were *TRAF3* (TNF Receptor-Associated factor 3; *P* = 2.1 × 10^−4^); *MAP2K1* (Mitogen-Activated Protein Kinase Kinase 1; *P* = 5.7 × 10^−3^); *SOS1;* (*P* = 0.019); *SHC1* (Src Homology two Domain Containing Transforming Protein 1; *P* = 0.027); *CREB5* (CAMP Responsive Element Binding Protein 5; *P* = 0.022); *NFKB1* (Nuclear factor of Kappa Light Polypeptide Gene Enhancer in B-cells 1; *P* = 0.025); and *BRAF* (B-Raf Proto-Oncogene, Serine/Threonine Kinase; *P* = 0.032). The *ERK1 ERK2 MAPK pathway* overlaps with *p130Cas linkage to MAPK signaling for integrins* pathway by two genes: *GRB2* and *SOS1* (Fig. 2).

The *MAP3K4* gene, in which we had observed SNP rs144240142 to be associated with Stage A endometriosis, is part of two pathways arising from the ST database: the *p38 MAPK pathway* and the c-Jun amino-terminal protein kinase (*JNK) MAPK pathway*. The *p38 MAPK pathway* overlaps with the *ERK1 ERK2 MAPK pathway* by 10 genes (*ATF1, CREB1, CREB3, CREB5, EEF2K, EIF4E, MAPK1, MKNK1, MKNK2, NFKB1*), while the *JNK MAPK pathway* overlaps by two genes (*DUSP4, SHC1*) (Fig. 2).

Other pathways significantly enriched (FDR-*P* and Bonferroni-*P* < 0.05) in GWAS results for Stage A endometriosis were (Table II) *TRKA receptor* (SA) and *PIP3 signaling in cardiac myocytes* (SIG). *G alpha S pathway* (ST), *Phosphoinositide three kinase pathway* (ST), *Differentiation pathway in PC12 cells* (ST), *B cell receptor complexes* (SA), *Insulin receptor pathway in cardiac myocytes* (SIG) and *PTEN pathway* (SA) were significant at FDR < 0.05 but not after stringent Bonferroni correction (see Materials and Methods).

For Stage B analysis, two pathways were significantly enriched (FDR-*P* and Bonferroni-*P* < 0.05): *ECM glycoproteins* (*P* = 1.8 × 10^−3^, FDR-*P* = 7.1 × 10^−3^, Bonferroni-*P* = 0.018) including 196 genes defined by Matrisome Project gene sets (http://web.mit.edu/hyneslab/matrisome/) (Naba *et al*., 2012), and *Core matrisome* comprising 275 genes encoding all known ECM glycoproteins, collagens and proteoglycans (*P* = 1.4 × 10^−3^, FDR-*P* = 0.013, Bonferroni-*P* = 0.014). The *ECM glycoproteins* gene set is a subset of the *Core Matrisome* and thus many of the genes enriched in the endometriosis GWAS overlap. Notably, this includes *FN1*, *FGG* and *FGA*, which are also part of the Reactome *MAPK*-related pathways associated with all endometriosis (Fig. 2).

Lastly, we set out to investigate to what extent pathways investigated previously in hypothesis-based candidate gene studies were highlighted by the hypothesis-free GWAS analyses. Among the 122 candidate genes investigated previously (see Materials and Methods), there was significant over-representation of investigation of 16 pathways (Table III), the top three being: *Interleukin signaling pathway* (*P* = 2.3 × 10^−12^), *Apoptosis signaling pathway* (*P* = 9.7 × 10^−9^) and *Gonadotropin releasing hormone receptor pathway* (*P* = 1.2 × 10^−6^). Others covered inflammation-related pathways, cellular homeostatic signaling, thrombolysis, angiogenesis and steroid biosynthesis. Only 2/122 genes were part of a pathway that was highlighted by our GWAS pathway analysis: *NFKB1* in ERK1 ERK2 MAPK pathway and *CDKN1B* in PIP3 signaling in cardiac myocytes (Table III). The results showed that of the pathways enriched in GWAS analyses only *Insulin/IGF pathway* with MAPK cascade and *PI3 kinase pathway* featured in the pathways explored in hypothesis-driven candidate gene association studies conducted to date, but others did not.

**Table III:** Most frequently investigated biological pathways for endometriosis through candidate gene studies, as defined by PANTHER database (July 2014)

PANTHER: Biological pathway	Candidate genes^a^
Interleukin signaling pathway (*n* = 95)	13 (*P* = 2.3 × 10^−12^)
Apoptosis signaling pathway (*n* = 113)	11 (*P* = 9.7 × 10^−9^)
Gonadotropin releasing hormone receptor pathway (*n* = 228)	12 (*P* = 1.2 × 10^−6^)
Inflammation mediated by chemokine/cytokine signaling (*n* = 233)	12 (*P* = 1.6 × 10^−6^)
p53 pathway (*n* = 81)	8 (*P* = 3.9 × 10^−6^)
Plasminogen activating cascade (*n* = 17)	5 (*P* = 9.4 × 10^−6^)
Insulin/IGF pathway-protein kinase B signaling cascade (*n* = 37)	6 (*P* = 1.4 × 10^−5^)
p53 pathway feedback loops 2 (*n* = 47)	5 (*P* = 1.3 × 10^−3^)
VEGF signaling pathway (*n* = 58)	5 (*P* = 3.6 × 10^−3^)
Angiogenesis (*n* = 152)	7 (*P* = 4.5 × 10^−3^)
Androgen/estrogene/progesterone biosynthesis (*n* = 10)	3 (*P* = 4.7 × 10^−3^)
Insulin/IGF pathway-mitogen-activated protein kinase kinase/MAP kinase cascade (*n* = 32)	4 (*P* = 6.1 × 10^−3^)
Alzheimer disease-presenilin pathway (*n* = 109)	6 (*P* = 6.5 × 10^−3^)
EGF receptor signaling pathway (*n* = 123)	6 (*P* = 0.013)
PI3 kinase pathway (*n* = 47)	4 (*P* = 0.027)

^a^Out of the 122 candidate genes 65 do not participate in any of the PANTHER pathways.

## Discussion

We have presented the first comprehensive analysis investigating biological pathways underlying endometriosis pathogenesis using genome-wide methodology. This analysis was based on GWAS results from all endometriosis cases, Stage A and Stage B cases vs. controls. Because the pathway results are based on genetic data, they provide strong evidence of causal involvement of these pathways in disease development and/or maintenance. In addition, we presented the first GWAS analysis for Stage A disease, further exploring the hypothesis of distinct causal pathophysiology on endometriosis subtypes.

The results confirm earlier reports that Stage A vs. Stage B have distinct genetic contributors (Painter *et al*., 2011; Nyholt *et al*., 2012; Rahmioglu *et al*., 2014), and highlight for the first time the role of *MAPK* signaling in its pathogenesis. The GWAS analysis of Stage A disease resulted in a novel associated variant, rs144240142, in an intronic region of *MAP3K4*, a gene that was also differentially expressed in endometrium from endometriosis cases and controls in our analysis of an independent data set (Tamaresis *et al*., 2014). This result is intriguing though it requires genotyping and replication in an independent data set. *MAP3K4* encodes for the protein MAPKKK4, which is part of the *p38* and *JNK MAPK* pathways. Subsequent pathway analysis revealed *ERK1 ERK2 MAPK* as the most significant pathway associated with Stage A endometriosis.

The pathway analysis for all cases of endometriosis revealed two pathways that were also linked to MAPK signaling: *Grb2-Sos provides linkage to MAPK signaling for Integrins* and *p130Cas linkage to MAPK signaling for integrins.* These pathways largely overlap (12 out of the 15 genes in each are the same), and reflect different routes through which integrins (adhesion molecules) can activate *ERK1 ERK2 MAPK* signaling. *Wnt signaling* was also implicated in all endometriosis, in line with previous reports of both single-SNP and pathway associations involving Wnt pathway genes (Painter *et al*., 2011; Nyholt *et al*., 2012; Sanchez *et al*., 2014; Rahmioglu *et al*., 2015a, 2015b), although the association was non-significant after stringent Bonferroni multiple-testing correction.

**Figure 3:**
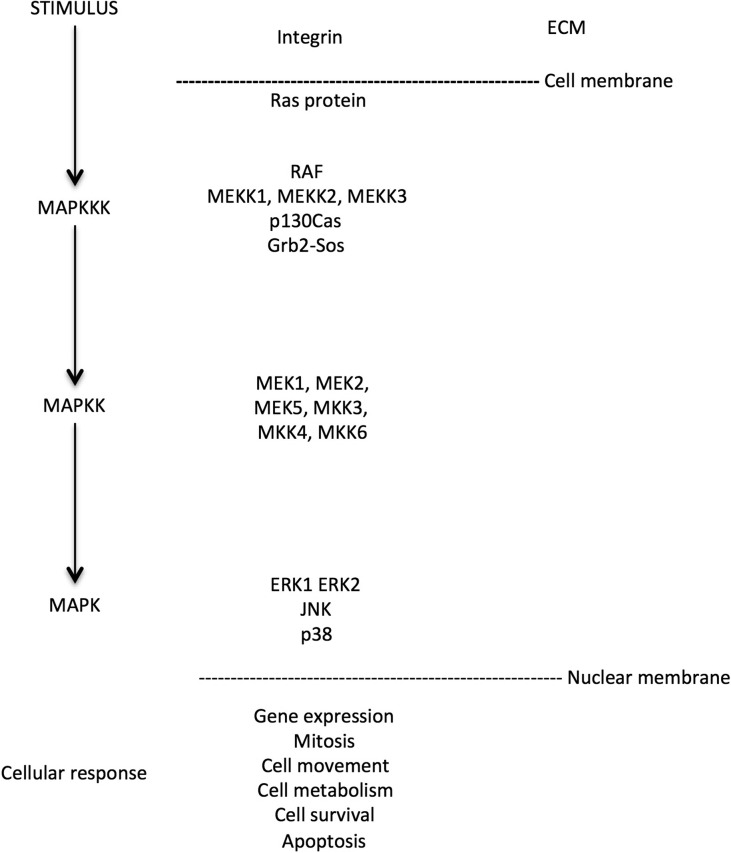
MAPK cascade and pathway components in mammalian cells. Adapted from Cabodi *et al*. (2010) and Zhang and Liu (2002).

MAPKs comprise a kinase enzyme family that add phosphate groups to other proteins to activate, triggering a cascade of downstream signaling reactions that are involved in many physiological processes, such as gene expression, mitosis, cell movement, metabolism, cell survival and apoptosis. The protein kinase cascade consists of enzyme components; MAPK kinase kinase (MAPKKK), MAPK kinase (MAPKK) and MAP kinase (MAPK), that are activated consecutively (Fig. 3). Extracellular stimuli activate the Grb2-Sos complex, which then promotes Ras protein activation and which in turn activate the cytosolic MAPK pathway that finally leads to intranuclear events and cellular responses. Three main MAPK classes are distinguished by their biological function: ERKs acting in the control of cell division; JNKs regulating transcription; and p38 MAPK playing important roles in immune response, cell survival and differentiation regulation. They are mostly activated by inflammatory cytokines and environmental stresses, while other stimuli include growth factors, neurotransmitters, steroid hormones, osmolarity and cell adherence (Widmann *et al*., 1999; Johnson and Lapadat, 2002).

A role for MAPK pathways has been recognized in a wide range of diseases, including cancers (Lei *et al*., 2014), obesity-associated insulin resistance (Hirosumi *et al*., 2002), ischemic heart disease (Bogoyevitch *et al*., 2004) and autoimmune diseases (Genovese, 2009). In endometriosis, MAPK signaling has been implicated previously, based on observed differential expression profiles and induction of cell proliferation in ectopic vs. eutopic endometrial cells in women with endometriosis vs. controls (Yotova *et al*., 2011; Li *et al*., 2013; Wu, *et al*., 2014). Gene expression within the *ERK MAPK* pathway has been suggested to be dysregulated in eutopic endometrium after experimental endometriosis induction in a baboon model (Afshar *et al*., 2013). However, these studies were unable to indicate whether these processes were causative to, or resulting from, the disease. Our results show that the association between *MAPK* signaling and endometriosis has a genetic basis and therefore likely reflects a causal mechanism, and furthermore, suggest that this association is limited to Stage A disease.

An intriguing mechanism through which *MAPK* signaling could be associated with Stage A endometriosis is through a role in increased sensitivity to pain, as *MAPK* pathways are differentially activated in neurons and glia in inflammatory pain and neuropathic pain conditions (Ji *et al*., 2009). A proportion of women with endometriosis have a neuropathic component involved in their pain mechanism (Whitaker *et al*., 2016). Interestingly, an animal model of endometriosis has shown p38 MAPK activation to play a role in this mechanism (Chen *et al*., 2015). Chronic pain patients appear less able to engage nociceptive pain processing and to produce endogenous pain inhibition (Tracey and Mantyh, 2007) and recent work suggests that dysfunction within this system may predispose an individual to develop chronic pain after an acute insult (De Felice *et al*., 2011). Thus, it may be that MAPK signaling is not associated with the presence of endometriosis *per se*, but with the experience of pain in women with Stage A disease, thereby increasing the likelihood of investigation and thus diagnosis. Our results show that associated variant rs144240142 resides in *MAP3K4*, part of the JNK and p38 MAPK classes, which overlap with the *ERK1 ERK2 MAPK* pathway that is genetically associated with Stage A disease. The implicated MAPK pathways act on the MAP kinase component level, more downstream in the cascade, close to the cell nucleus (Fig. 3). As these MAPK classes are functionally connected, they require a comprehensive functional investigation in relation to endometriosis.

The most significant pathways associated with Stage B (rAFS III/IV) disease were related to ECM and adhesion processes: the *Core Matrisome*, a large pathway containing 275 genes encoding all known ECM glycoproteins, collagens and proteoglycans that includes a sub-pathway, *ECM glycoproteins* containing 196 genes. Notably, the top genes associated with Stage B endometriosis in these pathways include *FN1*, *FGG* and *FGA* which are also part of the Reactome *integrin linkage to MAPK* pathways associated with all endometriosis. These pathways act on the MAPKKK component level, upstream in the cascade close to cellular membrane (Fig. 3). GWAS and replication analyses have shown that the association between genetic variants in *FN1*, encoding for fibronectin, and endometriosis is limited to Stage B disease (Pagliardini *et al*., 2013; Rahmioglu *et al*., 2014). Our pathway results confirm a key role of ECM glycoprotein biology limited to Stage B disease. This observation is interesting, because it is the pathological accumulation of ECM proteins that defines fibrosis—a common hallmark in Stage B (rAFS III/IV) disease.

The role of integrins and the ECM in endometriosis has long been postulated (Klemmt *et al*., 2007; Pitsos and Kanakas, 2009). Integrins are transmembrane adhesion receptors and construct bridges for cell–cell and cell–ECM interactions. Studies have shown that integrin expression, particularly on peritoneal mesothelial cells, indicates a potential attachment site for ectopic endometrial cells (Witz *et al*., 2000), while integrins present in endometriosis lesions have been shown to be of peritoneal origin (Hull *et al*., 2008). Aberrations in ECM have been documented in relation to endometriosis, but it is not clear whether the mesothelial layer is damaged thus exposing the ECM, or whether inherent changes in the ECM promote the adherence of primary endometrial cells. Our findings suggest the latter.

GWAS analysis is a powerful tool in uncovering associations between common genetic variants and complex diseases such as endometriosis (Manolio *et al*., 2009), but requires large sample sizes to enable detection of associated variants at ‘genome-wide significance’ (*P* < 5 × 10^−8^). For instance, for breast cancer the largest GWAS meta-analysis has included >55 000 cases to date, finding 67 GWAS variants together explaining 14% of heritability (Michailidou *et al*., 2013). Case numbers for endometriosis discovery GWAS have been <1/10 the size, and have discovered only eight variants that explain <4% of heritability (Rahmioglu *et al*., 2014). Pathway analysis provides a powerful approach to leverage GWAS variant data in their cumulative effects into genes and pathways, thus substantially reducing the statistical impact of multiple testing and superseding the agnostic approach by highlighting functional pathways involved in pathogenesis. Increased sample sizes for endometriosis GWAS analysis are needed to identify further genetic variants associated with disease pathology that will also add to the analysis power and robustness of the pathways identified for endometriosis.

Drawbacks of pathway analysis are (i) only variants in or near genes are assigned to genes so that a large proportion of GWAS evidence pertaining to intergenic variants is disregarded; (ii) reliance on the contents of pathway databases and on the available software tools within them. Better studied biological processes are more likely to be defined into (more detailed) pathways than less well-studied ones. In our analysis, this may have resulted in the relatively greater detail of the different *MAPK* pathways highlighted. Our results summarizing candidate gene studies conducted to date showed that these have largely focused on pathways that do not feature in our genome-wide results. This does not mean that these candidate-based pathways are irrelevant to endometriosis causation, but rather that more consideration should be given to *MAPK* regulated ECM and integrin involving processes and Wnt signaling as relatively under-investigated pathways, in terms of biological follow-up.

An important method for understanding the effects of variants on downstream molecular processes is their integration with transcriptomic and epigenomic data. However, such functional studies need to be conducted in disease-related tissues, such as endometrium for endometriosis. There are no genome-wide data sets available currently that associate genetic variants with gene expression levels (expression quantitative trait loci) in endometrium. Currently, none of the large-scale genomic profiling initiatives such as GTex (Consortium, 2013) and the NIHR Epigenome Roadmap (Skipper *et al*., 2015) include endometrium, and very limited endometrium annotation data are available in ENCODE (Consortium, 2012). The lack of genomic annotation data currently severely impedes the ‘translation’ of genetic signals associated with endometriosis into their downstream effects.

MAPK, and Wnt signaling, pathways have been targeted for drug discovery, mainly in the area of cancer (Sebolt-Leopold and Herrera, 2004), but the ubiquitous functional nature of these pathways represents considerable challenges for a non-malignant condition such as endometriosis. Protein kinase inhibitors targeting the *ERK1 ERK2 pathway* were observed to control deep infiltrating endometriosis progression (Ngo *et al*., 2010) and their role in endometriosis treatment has been explored in several studies (Santulli *et al*., 2015). To date, they present unacceptable adverse effects, including ovulation inhibition and teratogenicity, skin and gastrointestinal toxicities, weight loss, fatigue, hypertension and infections (Krajewska *et al*., 2015). Targeting the Wnt signaling pathway has similar challenges (Duchartre *et al*., 2016). This does not negate the potential for more specific targets to be identified in these pathways that limit unwanted consequences, or topical rather than systemic applications to be developed. More studies are needed in this area.

Our results offer further support for the hypothesis of at least partially distinct causal pathophysiology for minimal/mild (rAFS I/II) vs. moderate/severe (rAFS III/IV) endometriosis. Based on our results, we postulate that integrin-mediated MAPK activation plays a key causal role in the establishment of endometriotic lesions. Development of Stage I/II disease is further characterized by involvement of the intracellular *ERK1 ERK2 pathway* acting downstream in the MAP kinase cascade, whereas Stage III/IV disease is characterized by other extracellular processes leading to increased fibrogenesis.

## Supplementary data

Supplementary data are available at *Human Reproduction* online.

## Authors’ roles

O.U. and N.R. are the lead authors. K.T.Z. developed the idea for the study and proposed the hypothesis. O.U., N.R. and K.T.Z. conceived and designed the study. K.T.Z. and G.W.M. obtained funding. N.R., D.R.N., G.W.M. and K.T.Z. provided study materials and collected and collated data. O.U., N.R. and A.P.M. did the statistical analysis. O.U., N.R. and K.T.Z. analyzed and interpreted the data. O.U. and N.R. made initial drafts of Tables and figures, and drafted the manuscript. D.R.N., K.V., S.A.M., C.B., A.P.M. and G.W.M. critically revised the manuscript for important intellectual content. All authors read and approved the final version of the manuscript. K.T.Z. is the study guarantor.

## Funding

The genome-wide association data and WTCCC were generated through funding from the Wellcome Trust (WT084766/Z/08/Z, and 076113) and the National Health and Medical Research Council (NHMRC) of Australia (241944, 339462, 389927, 389875, 389891, 389892, 389938, 443036, 442915, 442981, 496610, 496739, 552485 and 552498). N.R. was funded by a grant from the Medical Research Council UK (MR/K011480/1). A.P.M. is a Wellcome Trust Senior Fellow in Basic Biomedical Science (WT098017).

## Conflict of interest

All authors declare there are no conflicts of interest.

## Supplementary Material

Supplementary Figure 1

Supplementary Figure 2

Supplementary Figure 3

Supplementary Table 1

Supplementary Table 2
