# Supplementary material for: Genome-wide genetic analyses highlight mitogen-activated protein kinase (MAPK) signaling in the pathogenesis of endometriosis
Source: Hum Reprod. 2017 Feb 9;32(4):780–93. doi: 10.1093/humrep/dex024 (PMC5400041; doi:10.1093/humrep/dex024)
Supplement: Supplementary Figure 1 [file dex024suppl_figure1.pdf]

Human Reproduction, 2017
doi:10.1093/humrep/dex024

human reproduction

SUPPLEMENTARY DATA

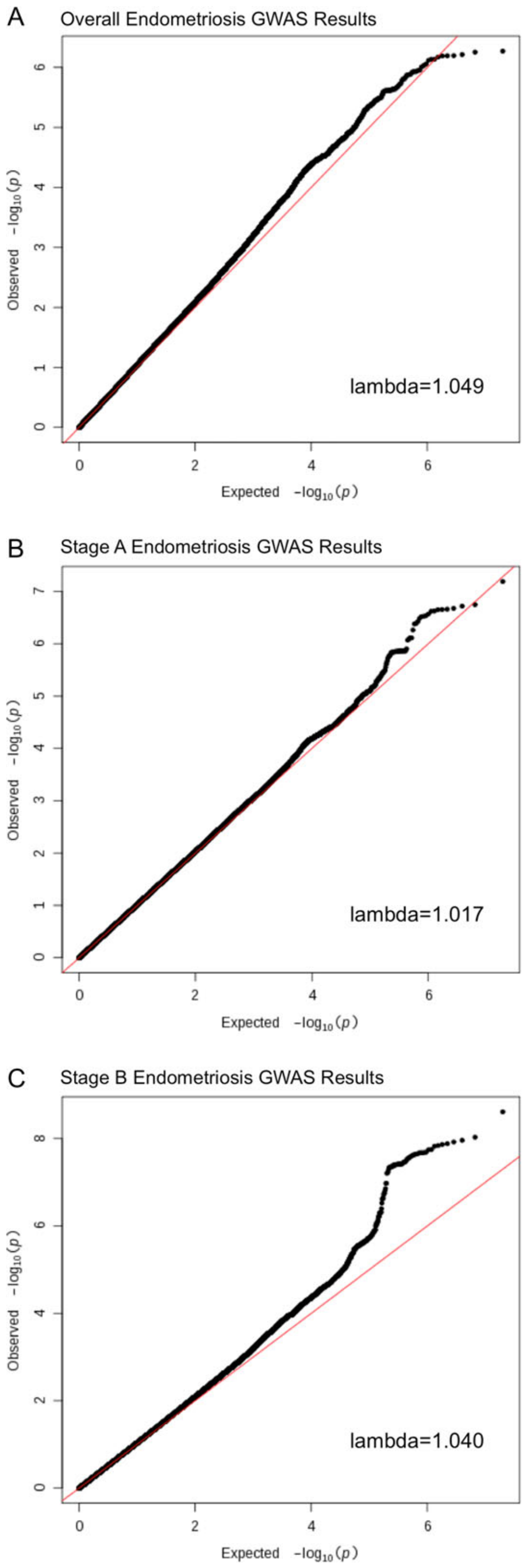

Supplementary Figure S1 QQ plots assessing genomic inflation of observed vs. expected genome-wide association study (GWAS) *P*-values for (**A**) overall endometriosis results, (**B**) Stage A endometriosis results and (**C**) Stage B endometriosis results.
