# Supplementary material for: Genome-wide genetic analyses highlight mitogen-activated protein kinase (MAPK) signaling in the pathogenesis of endometriosis
Source: Hum Reprod. 2017 Feb 9;32(4):780–93. doi: 10.1093/humrep/dex024 (PMC5400041; doi:10.1093/humrep/dex024)
Supplement: Supplementary Figure 2 [file dex024suppl_figure2.pdf]

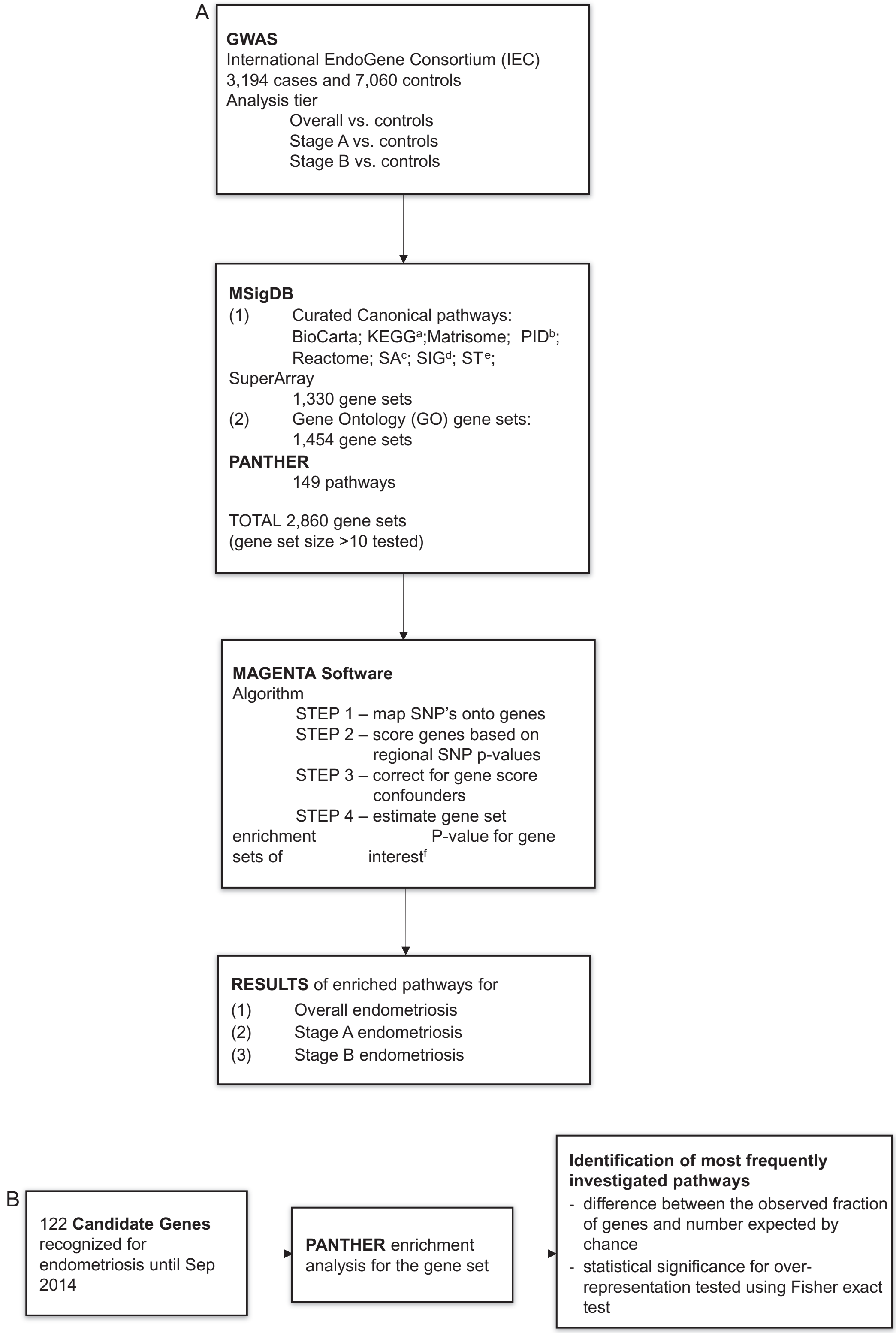

Supplementary Figure S2 Study designs for (**A**) the GWAS pathway analysis on endometriosis and (**B**) identification of pathways that have been investigated indirectly through candidate gene association studies for endometriosis. [a]Kyoto Encyclopedia of Genes and Genomes; [b]Pathway Interaction Database; [c]SigmaAldrich; [d]Signaling Gateway; [e]Signal Transduction; [f]Segre *et al. PLoS Genetics*, 2010.
