# Supplementary material for: Genome-wide genetic analyses highlight mitogen-activated protein kinase (MAPK) signaling in the pathogenesis of endometriosis
Source: Hum Reprod. 2017 Feb 9;32(4):780–93. doi: 10.1093/humrep/dex024 (PMC5400041; doi:10.1093/humrep/dex024)
Supplement: Supplementary Figure 3 [file dex024suppl_figure3.pdf]

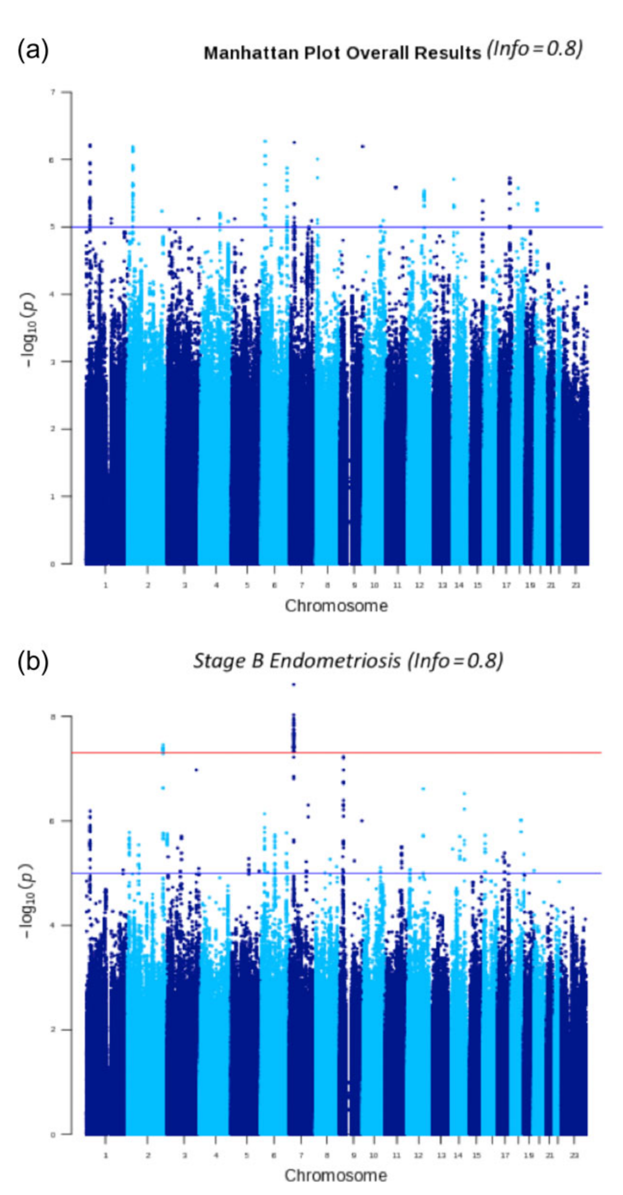

Supplementary Figure S3 Manhattan plots of association of single-nucleotide polymorphisms (SNPs) with (**a**) all and (**b**) stage B endometriosis in the GWAS. Red horizontal line marks the genome-wide significance ($P < 5 \times 10^{-8}$), and blue line marks nominal significance ($P < 5 \times 10^{-6}$) .
