# Supplementary material for: Genome-wide genetic analyses highlight mitogen-activated protein kinase (MAPK) signaling in the pathogenesis of endometriosis
Source: Hum Reprod. 2017 Feb 9;32(4):780–93. doi: 10.1093/humrep/dex024 (PMC5400041; doi:10.1093/humrep/dex024)
Supplement: Supplementary Table 1 [file dex024suppl_table1.pdf]

Supplementary Table SI **Study population for genome-wide association study (GWAS).**

The International Endogene Consortium, IEC	Patients (*n*)		Patients (%)
	UK	Australia	
Surgically confirmed cases			
Stage A (I–II)	329	1357	52.7
Stage B (III–IV)	454	910	42.7
Unclassified	141	3	4.6
Total	924	2270	100
Controls	5190	1870	

Endometriosis cases were classified according to the revised American Fertility Society (rAFS) classification through assessment of surgical records.
