# Supplementary material for: Genome-wide genetic analyses highlight mitogen-activated protein kinase (MAPK) signaling in the pathogenesis of endometriosis
Source: Hum Reprod. 2017 Feb 9;32(4):780–93. doi: 10.1093/humrep/dex024 (PMC5400041; doi:10.1093/humrep/dex024)
Supplement: Supplementary Table 2 [file dex024suppl_table2.pdf]

Supplementary Table SII Differential expression analysis in eutopic endometrium including 77 endometriosis cases and 71 controls.

GWAS analysis	Gene	Probe_ID*	Adj-*P*-Val	*P*-value	Beta	logFC
Overall GWAS Loci	*ID4*					
	Overall	229386_at	8.53×10^{-3}	1.41×10^{-3}	−1.458	−0.43
	Stage B	229386_at	0.011	8.00×10^{-4}	−0.897	0.54
	Stage A	229386_at	0.16	0.077	−4.973	0.29
	NFE2L3					
	Overall	204702_s_at	0.031	7.79×10^{-3}	−2.991	0.34
	Stage B	204702_s_at	0.24	0.091	−5.029	−0.27
	Stage A	204702_s_at	0.031	0.011	−3.289	−0.45
	WNT4					
	Overall	1556689_a_at	0.15	0.064	−4.795	−0.32
	Stage B	1556689_a_at	1	0.96	−6.440	-8.04×10^{-3}
	Stage A	1556689_a_at	2.30×10^{-3}	4.09×10^{-4}	−0.322	0.89
	LAMC3					
	Overall	219407_s_at	0.33	0.19	⁻5.651	−0.32
	Stage B	219407_s_at	0.93	0.84	−6.420	−0.057
	Stage A	219407_s_at	0.018	5.43×10^{-3}	−2.684	0.91
	CAPN14					
	Overall	1557321_a_at	0.51	0.36	−6.077	0.083
	Stage B	1557321_a_at	0.51	0.31	−5.926	−0.11
	Stage A					
	DEFA1					
	Overall	205033_s_at	0.54	0.40	−6.131	0.16
	Stage B	205033_s_at	0.59	0.40	−6.083	−0.18
	Stage A					
Stage A GWAS Loci	*MAP3K4*					
	Overall	216199_s_at	7.12×10^{-3}	1.11×10^{-3}	−1.242	0.60
	Stage B	216199_s_at	0.26	0.10	−5.122	−0.31
	Stage A	216199_s_at	3.76×10^{-4}	2.44×10^{-5}	2.310	−1.03
	CDC73					
	Overall	235196_at	3.97×10^{-4}	1.78×10^{-5}	2.591	0.67
	Stage B	235196_at	0.021	2.15×10^{-3}	−1.797	−0.53
	Stage A	235196_at	2.58×10^{-4}	1.21×10^{-5}	2.971	−0.88
	CSMD1					
	Overall	1553405_a_at	0.027	6.50×10^{-3}	−2.831	−0.15
	Stage B	1553405_a_at	0.061	0.011	−3.241	0.15
	Stage A	1553405_a_at	0.031	0.011	−3.267	0.15
	EMX2OS (Anti-sense)					
	Overall	230963_at	0.27	0.15	−5.439	−0.28
	Stage B	230963_at	0.74	0.57	−6.280	−0.12
	Stage A	230963_at	1.37×10^{-3}	1.96×10^{-4}	0.359	0.92
	ALCAM					
	Overall	201951_at	0.069	0.023	−3.922	0.56
	Stage B	201951_at	0.70	0.52	−6.234	−0.18
	Stage A	201951_at	1.36×10^{-3}	1.93×10^{-4}	0.372	−1.18

Continued

Supplementary Table SII *Continued*

GWAS analysis	Gene	Probe_ID*	Adj-*P*-Val	*P*-value	Beta	logFC
Stage B GWAS Loci	*NFE2L3*					
	See above					
	NAALADL2					
	Overall	1557998_at	0.64	0.51	−6.272	−0.021
	Stage B	1557998_at	0.75	0.59	−6.294	0.018
	Stage A					
	CDKN2BAS1					
	Overall	1557998_at	0.64	0.51	−6.272	−0.021
	Stage B	1557998_at	0.75	0.59	−6.292	0.018
	Stage A					
	FN1					
	Overall	214701_s_at	0.057	0.017	−3.694	−0.32
	Stage B	214701_s_at	0.015	1.26×10^{-3}	−1.312	0.54
	Stage A	214701_s_at	0.36	0.22	−5.779	−0.060
	NR2C1					
	Overall	204791_at	0.021	4.65×10^{-3}	−2.532	0.62
	Stage B	204791_at	0.33	0.15	−5.414	−0.33
	Stage A	204791_at	1.67×10^{-3}	2.62×10^{-4}	0.091	−1.06
	C14orf132					
	Overall	231859_at	3.80×10^{-5}	3.36×10^{-7}	6.337	0.82
	Stage B	231859_at	6.87×10^{-5}	1.79×10^{-7}	6.973	−0.98
	Stage A	231859_at	0.021	6.73×10^{-3}	−2.875	−0.53
	FOXP2					
	Overall	1555352_at	0.16	0.071	−4.869	−0.011
	Stage B	1555352_at	0.33	0.15	−5.407	6.79×10^{-3}
	Stage A	1555352_at	0.19	0.098	−5.163	9.18×10^{-3}
	WNT4					
	See above					
	ID4					
	See above					
	CDH20					
	Overall	210913_at	0.46	0.31	−5.972	-1.65×10^{-3}
	Stage B	210913_at	0.37	0.18	−5.543	2.64×10^{-3}
	Stage A					
	LAMC3					
	See above					

Of the 77 endometriosis cases, 48 are Stage B cases and 27 are Stage A cases. The expression analysis results including all endometriosis cases, only Stage B and only Stage A cases are presented (Tamaresis *et al.*, 2014). Adj-*P*-Val, Multiple-testing adjusted *P*-value (false discovery rate (FDR) corrected); *P*-value, nominal *P*-value; Beta, Beta-value, which is log-odds that the gene is differentially expressed, logFC: Log2-fold change in expression between cases and controls.

*Multiple probes map to some of the genes. Of these, the probe with lowest *P*-value is reported.
